# Association of Planning Target Volume with Patient Outcome in Inoperable Stage III NSCLC Treated with Chemoradiotherapy: A Comprehensive Single-Center Analysis

**DOI:** 10.3390/cancers12103035

**Published:** 2020-10-19

**Authors:** Monika Karin, Julian Taugner, Lukas Käsmann, Chukwuka Eze, Olarn Roengvoraphoj, Amanda Tufman, Claus Belka, Farkhad Manapov

**Affiliations:** 1 Department of Radiotherapy and Radiation Oncology, University Hospital, LMU Munich, 81377 Munich, Germany; Monika.Karin@med.uni-muenchen.de (M.K.); Julian.Taugner@med.uni-muenchen.de (J.T.); Chukwuka.Eze@med.uni-muenchen.de (C.E.); Olarn.Roengvoraphoj@med.uni-muenchen.de (O.R.); Claus.Belka@med.uni-muenchen.de (C.B.); Farkhad.Manapov@med.uni-muenchen.de (F.M.); 2 Comprehensive Pneumology Center Munich (CPC-M), German Center for Lung Research (DZL), Center for Lung Research (DZL), 81377 Munich, Germany; Amanda.Tufman@med.uni-muenchen.de; 3 German Cancer Consortium (DKTK), Partner Site Munich, 80336 Munich, Germany; 4 Division of Respiratory Medicine and Thoracic Oncology, Department of Internal Medicine V, Thoracic Oncology Centre Munich, LMU Munich, 81377 Munich, Germany

**Keywords:** NSCLC, multimodal treatment, stage III, survival, prognostic factor

## Abstract

**Simple Summary:**

Non-small cell lung cancer (NSCLC) in stage III is often inoperable and highly heterogeneous. The primary gross tumor volume is prognostically relevant in several types of cancer, including oral carcinoma, B-cell lymphoma, and sarcoma. The planning target volume (PTV), including the primary tumor and involved lymph node stations, can vary widely, and its prognostic value for stage III is unclear. We aimed to evaluate the impact of the PTV for overall survival (OS), progression-free survival, and loco-regional control in 122 consecutive patients treated with definitive chemoradiotherapy (CRT). Median follow-up for the entire cohort was 41.2 (range: 4–108) months; median overall survival (OS) reached 20.9 (95% CI: 14.5–27.3) months. In a multivariate analysis including age, gender, total radiation dose, and histology, PTV ≥ 700 ccm was found to be an independent prognostic factor for OS (hazard ratio (HR): 1.705, 95% confidence interval (CI): 1.071–2.714, *p* = 0.025). In conclusion, non-operable stage III NSCLC patients with PTV ≥ 700 ccm showed significantly detrimental outcomes after conventionally fractionated CRT. PTV should be considered as a stratification factor in multimodal clinical trials for inoperable stage III NSCLC.

**Abstract:**

Inoperable stage III non-small cell lung cancer (NSCLC) represents a highly heterogeneous patient cohort. Multimodal treatment approaches including radiotherapy have been the new standard of care, with promising outcomes. The planning target volume (PTV), including the primary tumor, involved lymph node stations and safety margins, can vary widely. In order to evaluate the impact of the PTV for overall survival (OS), progression-free survival (PFS) and loco-regional control, we analyzed retrospective and prospective data of 122 consecutive patients with inoperable stage III NSCLC treated with CRT. The majority of patients (93%) received a total dose ≥ 60 Gy and 92% of all patients were treated with concurrent or sequential chemotherapy. Median follow-up for the entire cohort was 41.2 (range: 3.7–108.4) months; median overall survival (OS) reached 20.9 (95% CI: 14.5–27.3) months. PTVs from 500 to 800 ccm were evaluated for their association with survival in a univariate analysis. In a multivariate analysis including age, gender, total radiation dose and histology, PTV ≥ 700 ccm remained a significant prognosticator of OS (HR: 1.705, 95% CI: 1.071–2.714, *p* = 0.025). After propensity score matching (PSM) analysis with exact matching for Union internationale contre le cancer (UICC) TNM Classification (7th ed.)T- and N-stage, patients with PTV < 700 ccm reached a median PFS and OS of 11.6 (95% CI: 7.3–15.9) and 34.5 (95% CI: 25.6–43.4) months vs. 6.2 (95% CI: 3.1–9.3) (*p* = 0.057) and 12.7 (95% CI: 8.5–16.9) (*p* < 0.001) months in patients with PTV ≥ 700 ccm, respectively. Inoperable stage III NSCLC patients with PTV ≥ 700 ccm had significantly detrimental outcomes after conventionally fractionated CRT. PTV should be considered as a stratification factor in multimodal clinical trials for inoperable stage III NSCLC.

## 1. Introduction

Lung cancer is the leading cause of cancer deaths worldwide, with an estimated 1.8 million deaths in 2018 [1,2]. Non-small cell lung cancer (NSCLC) accounts for about 85% of all lung cancer cases and is typically diagnosed at an advanced stage [3,4]. Inoperable stage III NSCLC represents a special entity due to the significant heterogeneity of the tumor and patient characteristics, as well as the multimodal approach required to treat it [5]. For these patients, concurrent chemoradiotherapy (CRT) remains the cornerstone of multimodal treatment [6,7,8,9,10,11]. According to pivotal trials concerning the intensification of the multimodal approach in inoperable stage III NSCLC, the planning target volume (PTV) was defined as an important factor for the quality of radiation delivery, treatment-related toxicity and patient outcome. In 2011, Salama et al. conducted a secondary analysis of the Cancer and Leukemia Group B (CALBG) 30,105 trial and found that a larger PTV and smaller total lung volume/PTV ratio were associated with increasing pulmonary toxicity in a univariate analysis [12]. Initial and long-term results of the Radiation Therapy Oncology Grou (RTOG) 0617 trial confirmed PTV as a prognosticator in inoperable stage III NSCLC treated with concurrent CRT in univariate and multivariate analyses [13,14]. Moreover, radiation therapy quality assurance within the PROCLAIM trial (Randomized Phase III Trial of Pemetrexed-Cisplatin or Etoposide-Cisplatin Plus Thoracic Radiation Therapy Followed by Consolidation Chemotherapy in Locally Advanced Nonsquamous Non-Small-Cell Lung Cancer) revealed that stage IIIB and PTV were associated with major violations in the delivered treatment plans [15].

Therefore, the purpose of the present study was to analyze the effects of PTV on patient outcome in inoperable stage III NSCLC treated with CRT and to define a volume cut-off that had the most impact on overall survival.

## 2. Patients and Methods

### 2.1. Patient Characterstics

This study included 122 consecutive patients who received concurrent or sequential conventionally fractionated CRT as part of a multimodal approach for stage IIIA/B (UICC 7th edition) NSCLC between 2011 and 2018 (prior to Durvalumab approval). An institutional review board (IRB), including the local ethics committee, approved this analysis (approval number: 17-230). Patients treated between January 2011 and December 2015 were included retrospectively and informed consent specifically for the retrospective part was not required by the IRB. Starting from January 2016, all patients were included prospectively and gave their informed consent.

Prior to the actual treatment, patient characteristics including tobacco consumption, performance status according to Eastern Cooperative Oncology Group (ECOG) and patients’ comorbidities were assessed. The majority of patients (96.7%) received a positron emission tomography (PET) computed tomography (CT) scan for treatment-planning. Screening for brain metastases was performed prior to treatment in all patients with cranial contrast-enhanced magnetic resonance imaging (MRI) in 54 (44.3%) patients and cranial contrast-enhanced CT in 64 (55.7%) patients. All patients underwent pulmonary function testing and routine blood testing in order to evaluate liver and kidney function as well as complete blood cell count. Treatment was discussed at multidisciplinary tumor boards with experienced thoracic surgeons classifying the tumors as unresectable. All patients with an ECOG performance status ≥ 2, poor lung function (diffusing capacity of the lung for carbon monoxide (DLCO) < 40%, forced expiratory volume in 1 second (FEV1) < 1 L or on long-term oxygen supply), total radiation therapy (RT) dose ≤ 54 Gy and TNM-stage other than stage III, were excluded.

### 2.2. Chemoradiotherapy

All patients were planned and treated between 2011 and 2018 at one tertiary cancer center. Based on conventional planning-CT as well as PET-CT scans in the treatment position, thoracic radiation therapy (TRT) was planned and carried out in the supine position and arms overhead using WingSTEP^TM^ (Innovative Technologie Völp, Innsbruck, Austria). In all cases, the target volumes were defined according to an in-house standard which is in close accordance to the later published European Society for Therapeutic Radiology and Oncology Advisory Committee in Radiation Oncology Practice (ESTRO-ACROP) guidelines [16]. If patients received induction chemotherapy, the residual primary tumor volume was delineated and cranio-caudal dimensions of clinical target volume (CTV) included initially involved lymph-node stations. Tumor motion management protocol was not routinely performed. PTV margins were 6 mm axial and 9 mm cranio-caudal beyond the CTV. Conventionally fractionated TRT was administered to the primary tumor and the involved lymph nodes with a median cumulative radiation dose of 66 Gy. Radiation delivery was performed using a linear accelerator (LINAC) with megavoltage capability of 6–15 MV with either 3D-CRT in 49 (40%) patients, intensity-modulated radiotherapy (IMRT, step and shoot, 38 patients) or volumetric modulated arc therapy (VMAT, 35 patients) in 73 (60%) patients. Image-guidance was performed with a cone-beam CT twice a week.

### 2.3. Patient Follow-Up

CT or PET-CT scans, routine complete blood work, lung function testing and clinical examinations were performed every 3 months in the first two years after therapy and twice yearly thereafter. Based on radiographic findings including CT, PET-CT or MRI, local and loco-regional progression (LP) along with distant metastases (DM) were calculated. Cytological or histological specimens to confirm disease progression were not obligatory. Median follow-up was calculated as the median time to loss or end of follow-up after the last day of radiotherapy in patients who were not documented as deceased. Progression-free survival (PFS) was defined as the time from the end of radiotherapy until disease progression or death. Overall survival (OS) was calculated from the end of radiotherapy until death. Regional recurrence was defined as progression/relapse in the ipsilateral lung or mediastinal/hilar lymph nodes.

### 2.4. Statistical Analysis

All statistics were performed using SPSS version 25 (IBM, Armonk, NY, USA). Univariate analysis was performed based on a comprehensive review of literature for PFS and OS with following parameters: age, gender, T- and N-stage, histology, RT dose and different PTV sizes between 500 ccm and 900 ccm. Multivariate analysis using Cox regression was carried out with PTV ≥ 600 ccm and ≥700 ccm as the two significant PTV values and other parameters showing a trend in the univariate analysis. Thereafter, we applied propensity score matching (PSM) using the R plug-in for IBM SPSS 25 [17,18,19,20,21,22,23,24] and performed an additional sensitivity analysis with exact matching of T- and N-stage.

## 3. Results

### 3.1. Patient and Tumor Characteristics

A summary of patient and tumor characteristics of the entire cohort, as well as the retrospectively- and prospectively-assessed subgroups, is shown in Table 1. The entire cohort consisted of 122 consecutive NSCLC patients with inoperable stage IIIA/B disease (UICC 7th edition stage) treated before Durvalumab approval. All patients received conventionally fractionated TRT. Median age was 68.5 with 81 (66.4%) patients older than 65 years. Forty-one (33.6%) were female and 81 (66.4%) male. On pre-treatment staging, 13 (10.7%), 20 (16.4%), 33 (27.0%) and 56 (45.9%) had T1, T2, T3 and T4 disease, and 15 (12.3%) N0, 9 (7.4%) N1, 44 (36.1%) N2 and 54 (44.3%) N3 disease, respectively. In the histological evaluation, 59 (48.4%) patients had squamous cell carcinomas (SCC), 52 (42.6%) had adenocarcinomas (AC) and in 11 (9.0%) patients, the tumor was classified as not otherwise specified (NOS). One hundred and thirteen (93%) patients received radiotherapy to a total dose ≥ 60 Gy (median total dose: 66 Gy; range 60–70 Gy). Concurrent CRT was delivered in 97 (79.5%) patients and 15 (12.3%) patients received sequential CRT. Ten (8.2%) patients were treated with TRT alone. Seventy-one patients (58.2%) received intravenous cisplatin at a dose of 20 mg/m^2^ on days 1–4 and oral vinorelbine (Navelbine) 50 mg/m^2^ on days 1, 8, and 15, every four weeks for two courses according to the German Intergroup Lung Trial (GILT) study [25]. The median follow-up for the entire cohort was 41.2 months (range: 3.7–108.4); median PFS and OS were 7.1 (95% CI: 5.9–8.4) and 20.9 (95% CI: 14.5–27.3), respectively.

### 3.2. Univariate and Multivariate Analysis

Patients older than 65 years had a median OS of 20.1 (95% CI: 14.0–26.2) vs. 25.5 (95% CI: 6.7–44.3) months (*p* = 0.066). Female patients had a median OS of 31.2 (95% CI: 24.1–38.3) vs. 16.3 (95% CI: 9.6–23.0) months for men (*p* = 0.022). Median OS/PFS was 20.6/17.6, 15.4/7.1, 12.9/6.4 and 25.5/6.6 months for patients with T1, T2, T3 and T4 disease, respectively (*p* = 0.753/0.330). For patients with N0, N1, N2 and N3 disease, median OS/PFS was 32.9/9.3, 23.4/7.2, 20.6/6.9 and 17.6/6.9 (*p* = 0.582/0.591), respectively. Patients with AC, SCC and NOS had a median OS/PFS of 27.2/7.4, 19.9/7.1, 12.7/5.6 months (*p* = 0.091/0.636), respectively. Patients irradiated to a total dose of at least 60 Gy had a longer overall survival of 23.1 (95% CI: 16.3–29.9) vs. 6.6 (95% CI: 5.7–7.5) months than others (*p* = 0.079). However, a total dose of ≥60 Gy was not a prognosticator of improved PFS (*p* = 0.352). PTV as a continuous variable showed a strong association with OS (*p* < 0.001). A significant correlation between PTV and patient outcome in the univariate analysis was demonstrated for PTV < 600 ccm and PTV < 700 ccm, whereas PTV 500 ccm, 800 ccm and 900 ccm showed no significant association with outcome.

For PTV < 600 ccm (*n* = 36, 29.5%), median OS was 34.5 (95% CI: 18.5–50.5) vs. 14.8 (95% CI: 8.0–21.6) months (*p* = 0.022) and median PFS was 8.2 (95% CI: 2.5–13.8) vs. 6.4 (95% CI: 4.5–8.2) months (*p* = 0.220).

**Figure 1:**
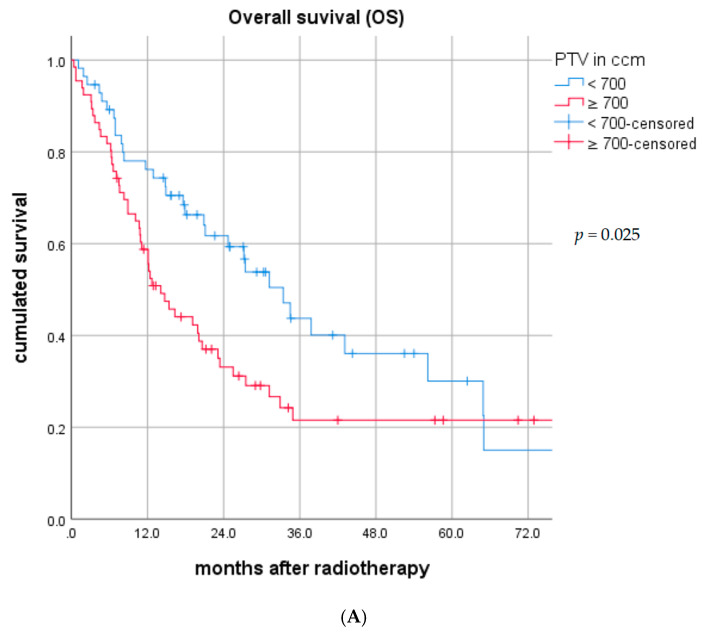

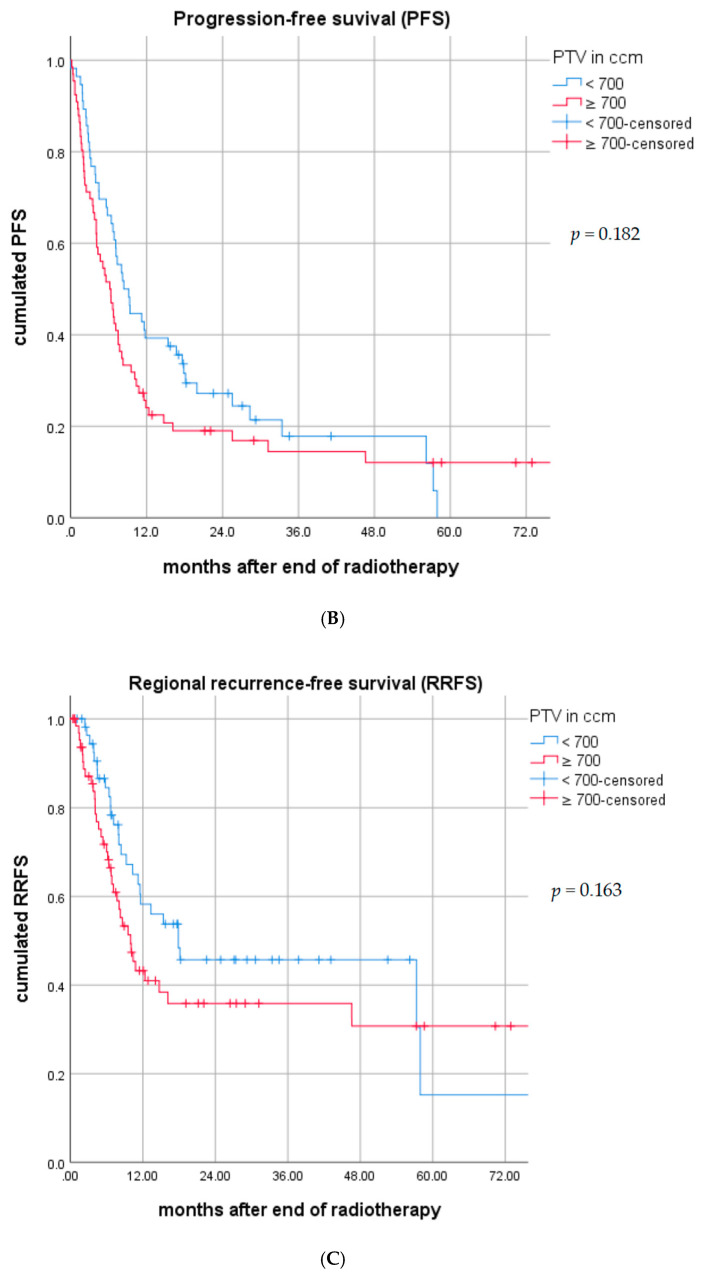
(**A**). Overall survival (OS) in the entire cohort by planning target volume (PTV) < 700 ccm vs. ≥700 ccm. (**B**) Progression-free survival (PFS) in the entire cohort by PTV < 700 ccm vs. ≥700 ccm. (**C**) Regional recurrence-free survival (RRFS) in the entire cohort by PTV < 700 ccm vs. ≥700 ccm.

For PTV < 700 ccm (*n* = 56, 45.9%), median OS was 33.4 (95% CI: 24.8–42.0) vs. 14.1 (95% CI: 9.7–18.5) months (*p* = 0.025) (Figure 1A) and median PFS was 8.4 (95% CI: 5.9–10.8) vs. 6.2 (95% CI: 4.1–8.2) months (*p* = 0.182) (Figure 1B). The median regional recurrence-free survival (RRFS) was 17.9 (95% CI: 0.0–43.2) vs. 10.0 (95% CI: 7.2–12.7) months (*p* = 0.163) in patients with PTV < 700 ccm vs. PTV ≥ 700 ccm, respectively (Figure 1C).

Multivariate analysis was performed separately for PTV ≥ 600 ccm and ≥700 ccm, as well as parameters showing a trend in the univariate analysis (*p* < 0.1) including age (≥65 years), gender, total dose of radiotherapy < 60 Gy and histology using Cox regression. For PTV ≥ 600 ccm, the hazard ratio (HR) was 1.715 (95% CI: 1.017–2.890, *p* = 0.043) and for ≥700 ccm, a HR of 1.705 (95% CI: 1.071–2.714, *p* = 0.025) was reached. It was shown that PTV ≥ 700 ccm was significant in the multivariate analysis.

Based on this result, all further calculations were carried out with PTV ≥ 700 ccm as a cut-off. Other parameters in the multivariate analysis with PTV ≥ 700 ccm showed the following results: for patients ≥ 65 years, the HR for death was 1.570 (95% CI: 0.945–2.609, *p* = 0.082); for male patients, the HR was 1.462 (95% CI: 0.896–2.387, *p* = 0.129); for total dose of radiotherapy < 60 Gy, the HR was 1.914 (95% CI: 0.863–4.246; *p* = 0.110); and for histology of SCC or NOS, the HR was 1.411 (95% CI: 0.983–2.026, *p* = 0.062). PTV ≥ 700 ccm was only a significant prognosticator for patients with SCC; median OS was 18.0 (95% CI: 11.8–24.2) vs. 35.4 (95% CI: 25.4–45.4) months (*p* = 0.010). For patients with AC, median OS was 43.5 (95% CI: 27.0–59.9) vs. 48.1 (95% CI: 30.7–65.5) months (*p* = 0.244).

### 3.3. PSM Analysis with Parameters Showing a Trend in Univariate Analysis

Patients with PTV < 700 ccm were matched at a 1:1 ratio to patients with ≥700 ccm. Propensity score (PS) matching was carried out with the parameters showing a trend in the univariate analysis (age, gender, RT total dose ≥60 Gy and histology) by nearest neighbor matching. The matched cohort consisted of 86 patients. In the subgroup with PTV < 700 ccm, there were 5 (11.6%), 8 (18.6%), 9 (20.6%) and 21 (48.8%) patients with T1, T2, T3 and T4 disease and 7 (16.3%), 4 (9.3%), 22 (51.2%) and 10 (23.3%) patients with N0, N1, N2 and N3 disease, respectively. In the subgroup with PTV ≥ 700 ccm, there were 1 (2.3%), 8 (18.6%), 13 (30.2%) and 21 (48.8%) patients with T1, T2, T3 and T4 disease and 4 (9.3%), 3 (7.0%), 8 (18.6%) and 28 (65.1%) patients with N0, N1, N2 and N3 disease, respectively. The median follow-up of the PSM cohort reached 44.3 months (range: 3.7–108.4); median OS of all matched patients was 19.9 (95% CI: 12.0–27.8) and median PFS was 7.1 (95% CI: 6.2–8.1) months. Patients with PTV < 700 ccm vs. ≥700 ccm had a median OS of 27.4 (95% CI: 15.2–39.6) vs. 12.4 (95% CI: 8.7–16.1) months (*p* = 0.009) (Figure S1A). Six, 12 and 24-month OS rates were 90.2% vs. 81.4%, 73.2% vs. 57.1% and 52.9% vs. 23.1% for PTV < 700 ccm vs. ≥ 700 ccm, respectively.

In the PTV < 700 ccm subgroup, the median PFS was 7.4 (95% CI: 4.4–10.3) months vs. 6.9 (95% CI: 5.2–8.6) months (*p* = 0.320) in patients with PTV ≥ 700 ccm (Figure S1B). Six, 12 and 24-month PFS-rates were 62.8% vs. 58.1%, 37.2% vs. 21.4% and 15.8% vs. 12.5% for PTV < 700 ccm vs. ≥700 ccm, respectively.

The median loco-regional recurrence-free survival (RRFS) was 17.9 (95% CI: 13.8–21.9) vs. 10.0 (95% CI: 7.6–12.4) months (*p* = 0.255) in patients with PTV < 700 ccm vs. ≥ 700 ccm (Figure S1C). We could not observe a difference in out-of-field recurrence (*p* = 0.768), whereas a trend in in-field-recurrence with better outcome for PTV < 700 ccm was revealed (*p* = 0.051).

It was confirmed that PTV 700 ccm is a significant prognostic factor for patients with SCC only. Patients with PTV < 700 ccm vs. PTV ≥ 700 ccm had an OS of 35.2 (95% CI: 23.8–46.4) vs. 16.8 (95% CI: 10.7–22.8) months (*p* = 0.014).

### 3.4. Additional PSM Analysis with Exact T- and N-Stage Matching

Patients with PTV < 700 ccm were matched at a 1:1 ratio to patients with PTV ≥ 700 ccm. To each patient with PTV < 700 ccm, one corresponding patient with exactly the same T- and N-stage was matched. The T/N-matched cohort consisted of 58 patients. A summary of patient and tumor characteristics is shown in Table 2. Both subgroups consisted of five (17.2%), six (20.7%), nine (31.0%) and nine (31.0%) patients with T1, T2, T3 and T4 disease and 5 (17.2%), 3 (10.3%), 11 (37.9%) and 10 (34.5%) patients with N0, N1, N2 and N3 disease, respectively. In the subgroup with PTV < 700 ccm, there were 21 (72.4%) patients with age ≥65, 16 (55.2%) males, 16 (55.2%) with SCC or NOS and 28 (96.6%) patients with total dose of ≥60 Gy. In the subgroup with PTV ≥ 700 ccm, there were 24 (82.8%) patients with age ≥65, 23 (79.3%) males, 16 (55.2%) with SCC or NOS and 28 (96.6%) patients with total radiotherapy dose of ≥60 Gy. The median follow-up of the T/N-matched cohort reached 44.3 months (range: 3.7–96.0); median OS was 24.7 (95% CI: 15.2–34.2) and median PFS was 8.2 (95% CI: 6.0–10.5) months.

**Figure 2:**
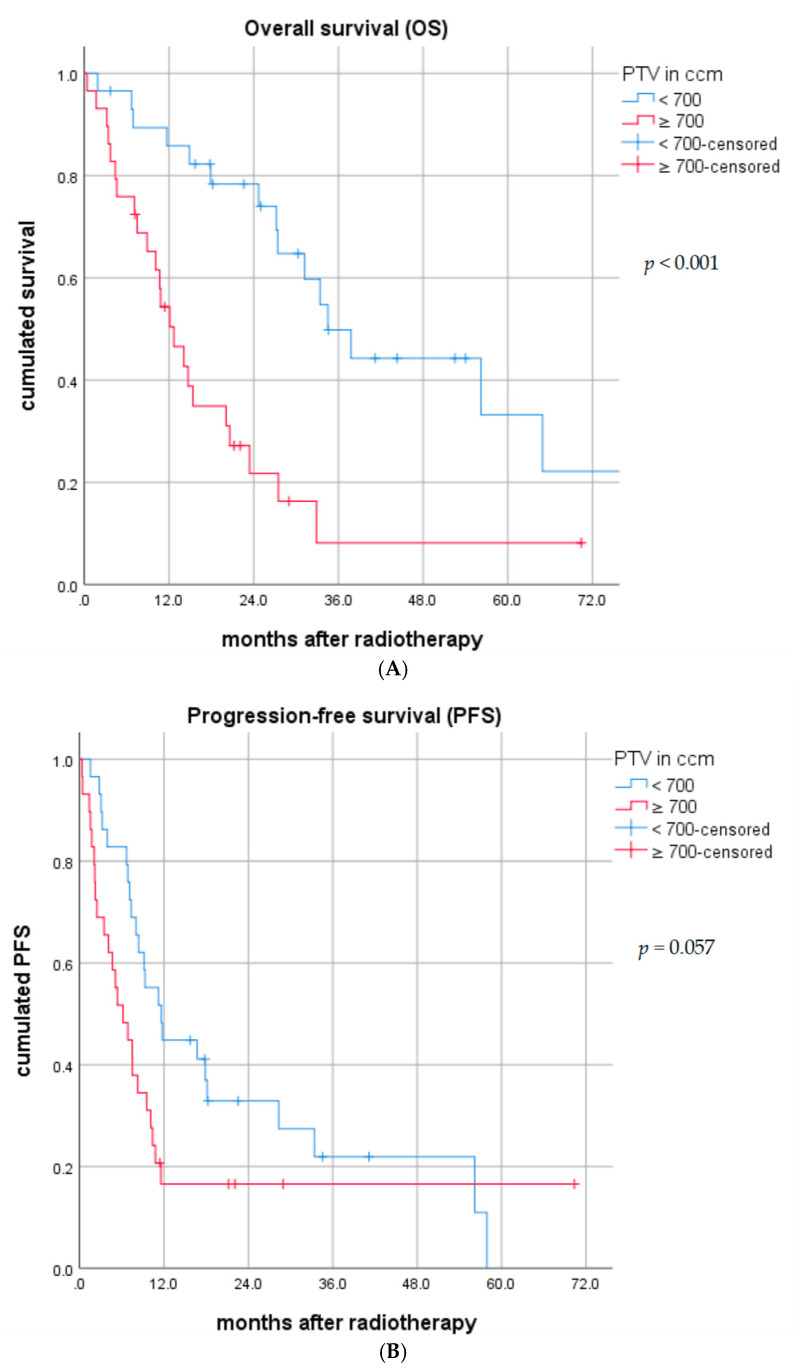

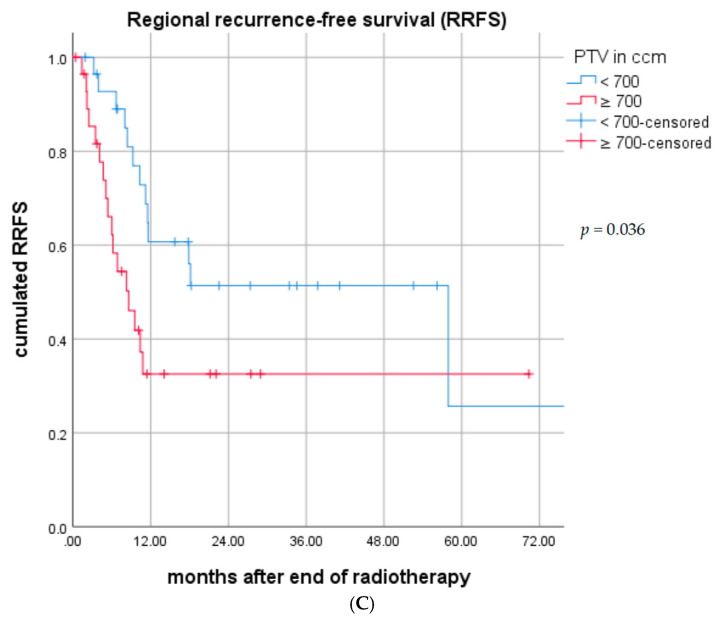
(**A**) Overall survival (OS) in the T/N-exact matched cohort by PTV < 700 ccm vs. ≥700 ccm. (**B**) Progression-free survival (PFS) in the T/N-exact matched cohort by PTV < 700 ccm vs. ≥700 ccm. (**C**) Regional recurrence-free survival (PFS) in the T/N-exact matched cohort by PTV < 700 ccm vs. ≥700 ccm.

In the T/N-matched patients with PTV < 700 ccm vs. ≥700 ccm, a median OS of 34.5 (95% CI: 25.6–43.4) vs. 12.7 (95% CI: 8.5–16.9) months (*p* < 0.001) was reached (Figure 2A). The 6, 12 and 24-month OS rates were 96.4% vs. 72.4%, 85.7% vs. 51.8% and 75.0% vs. 16.0%, respectively. In the PTV < 700 ccm subgroup, the median PFS was 11.6 (95% CI: 7.3–15.9) months vs. 6.2 (95% CI: 3.1–9.3) months (*p* = 0.057) in patients with PTV ≥ 700 ccm (Figure 2B). The 6, 12 and 24-month PFS rates were 82.8% vs. 51.7%, 44.8% vs. 14.3% and 24.0% vs. 7.7%, respectively. The median regional recurrence-free survival (RRFS) was 57.9 (95% CI: 9.1–106.7) vs. 2.0 (95% CI: 4.6–12.6) months (*p* = 0.036) in patients with PTV < 700 ccm vs. PTV ≥ 700 ccm, respectively (Figure 2C).

Compared to the entire cohort, PTV ≥ 700 ccm was revealed to be a significant prognostic factor independent of tumor histology in the T- and N-matched cohort. Patients with SCC and PTV < 700 ccm vs. ≥ 700 ccm had an OS of 24.7 (95% CI: 1.7–47.8) vs. 14.7 (95% CI: 6.8–22.6) months (*p* = 0.049) whereas patients with AC and PTV < 700 ccm vs. PTV ≥ 700 ccm had an OS of 37.8 (95% CI: 27.2–52.5) vs. 12.1 (95% CI: 6.9–17.3) months (*p* = 0.001).

## 4. Discussion

The aim of the present study was to provide a comprehensive analysis of the role of PTV (including the primary tumor and involved lymph node stations) in inoperable stage III NSCLC treated with CRT. Analyzed data were retrospectively and prospectively collected at a single tertiary cancer center. One hundred twenty-two consecutive cases with a total radiation dose to the primary tumor of at least 54 Gy were evaluated.

The main conclusion of the analysis is that PTV is continuously associated with patient outcome after the completion of CRT. Furthermore, the univariate, multivariate and PSM analyses performed demonstrated that PTV ≥ 700 ccm had the greatest impact on patient survival (PFS, OS) and may be considered as a stratification factor in clinical trials for inoperable stage III NSCLC. According to the PSM analysis with exact T- and N-stage matching, a significant difference in OS and a clear trend for PFS was elucidated. Patients with PTV < 700 ccm had a 12-month PFS rate of 45% vs. only 14% in patients with PTV ≥ 700 ccm. More frequent in-field recurrences in patients with PTV ≥ 700 ccm were also documented (*p* = 0.051). Furthermore, patients with PTV < 700 ccm reached a median OS of 34.5 vs. only 12.7 (95% CI: 8.7–16.1) months in patients with PTV ≥ 700 ccm (*p* < 0.001).

In lung cancer, an increasing tumor volume is associated with a significant decline in patient outcome. More than a decade ago, Werner-Wasik et al. performed a secondary analysis of the Radiation Therapy Oncology Group 93–11 Phase I–II dose escalation study in inoperable NSCLC and revealed that patients with smaller (gross tumor volume (GTV) ≤ 45 cm^3^) tumors had a longer OS and PFS than patients with larger (GTV > 45 cm^3^) tumors. GTV was defined as a sum of the volumes of the primary tumor and involved lymph nodes; the analysis also found that dose escalation had no effect on patient outcome in the treated cohort [26].

Basaki et al. evaluated 71 patients with stage III NSCLC treated with definitive (chemo)radiation and reported that total tumor volume and primary tumor volume, but not nodal volume, significantly influenced OS [27]. In contrast, both nodal and primary tumor volumes were associated with OS and local control in patients with stage III NSCLC after CRT in a retrospective review from the Dana–Farber Cancer Institute [28]. A multicenter prospective observational study (Trans-Tasman Radiation Oncology Group (TROG) 99.05) on 509 eligible stage I–II NSCLC patients treated with definitive TRT demonstrated the complex relationship between tumor volume and survival. At first, a larger primary tumor volume was associated with shorter survival (HR = 1.060, 95% CI: 1.01–1.12, *p* = 0.029). However, once the effects of T- and N-stage were corrected for, the association waned (HR = 1.029, 95% CI: 0.96–1.10, *p* = 0.39). There was still evidence that a larger primary tumor volume, regardless of T- and N-stage, was associated with an increased risk of death in the first 18 months [29].

A retrospective analysis from Dehing-Oberije et al. on 270 consecutive patients with stage I–III NSCLC radically treated with (chemo) radiation also reported a prognostic role for both, i.e., volume of the primary tumor and involved nodes as well as number of positive lymph nodes stations [30]. According to the ESTRO-ACROP guidelines for locally advanced NSCLC, published in 2018, positive (involved) lymph node stations will be included in the CTV and thus also in the PTV [16]. To avoid methodical discrepancies, we analyzed the PTV which considered the total tumor volume itself, the clinical target volume with positive lymph node stations, as well as safety margins for potential patient positioning and setup errors.

Importantly, the results of our analysis are in close accordance with previously published data from Wiersma et al. Both are studies from high volume cancer centers that included inoperable stage III NSCLC patients treated with CRT. Furthermore, both analyses evaluated the role of PTV and found that 700 ccm as a cut off is important for patient outcome [31]. In contrast to Wiersma et al. however, we also evaluated PTV as a continuous variable. In addition, we tested different PTVs from 500 to 800 ccm and performed a PSM analysis with exact T- and N-stage matching to confirm its prognostic role. A short overview of studies confirming a prognostic role of PTV in NSCLC patients treated with conventionally fractionated CRT is provided in Table 3.

The results of our analysis suggest that for inoperable stage III NSCLC patients with PTV ≥ 700 ccm, the multimodal approach definitely needs to be further refined. The incorporation of immune checkpoint inhibition (CPI) into the treatment paradigm may play a special role in this group of patients. A secondary analysis of trials establishing CPI as a consolidation treatment after CRT in patients with PTV ≥ 700 ccm will be of particular importance. Also, a proof of novel neoadjuvant concepts including chemoimmunotherapy may be promising in this subgroup. Another important point will be the optimization of tumor motion control during CRT. The use of abdominal compression and deep inspiration breath hold, as well as the establishment of four-dimensional cone-beam CT technology for daily image guidance, will help to reduce positioning and setup errors.

Important limitations of the present analysis are its single-center design and lack of comprehensive toxicity data. Nevertheless, the analyzed cohort consists exclusively of patients with inoperable stage III NSCLC and the definition of PTV was based on the Fluorodeoxyglucose (FDG)-PET/CT in treatment position. In the absolute majority of patients, target volumes were defined according to the international guidelines (ESTRO-ACROP). Finally, a comprehensive statistical evaluation including PSM analysis with exact T- and N-stage matching was done to confirm the prognostic role of PTV.

## 5. Conclusions

The present study revealed that PTV (including the primary tumor and involved lymph node stations) is an important prognosticator in patients with inoperable stage III NSCLC treated with conventionally fractionated CRT. Patients with PTV ≥ 700 ccm represent a special subgroup with significantly lower loco-regional control, worse PFS and worse OS. We recommend evaluating PTV as an additional stratification factor in clinical trials of multimodal therapy in inoperable stage III NSCLC.

## Figures and Tables

**Table 1:** Patient and tumor characteristics of the entire cohort and both subgroups.

Parameter	Entire Cohort	Retrospective Subgroup	Prospective Subgroup
	*N* (%)	*N* (%)	*N* (%)
Total	122	86	36
Age, Years			
≥65	81 (66.4)	58 (67.4)	23 (63.9)
<65	41 (33.6)	28 (32.6)	13 (36.1)
Gender			
Male	81 (66.4)	54 (62.8)	27 (75.0)
Female	41 (33.6)	32 (37.2)	9 (25.0)
T-stage			
1	13 (10.7)	7 (8.1)	6 (16.7)
2	20 (16.4)	16 (18.6)	4 (11.1)
3	33 (27.0)	27 (31.4)	6 (16.7)
4	56 (45.9)	36 (41.9)	20 (55.6)
N-stage			
0	15 (12.3)	9 (10.5)	6 (16.7)
1	9 (7.4)	7 (8.1)	2 (5.6)
2	44 (36.1)	29 (33.7)	15 (41.7)
3	54 (44.3)	41 (47.7)	13 (36.1)
Histology			
Squamous cell carcinoma (SCC)	59 (48.4)	38 (44.2)	21 (58.3)
Adenocarcinoma (AC)	52 (42.6)	41 (47.7)	11 (30.6)
Not otherwise specified (NOS)	11 (9.0)	7 (8.1)	4 (11.1)
Radiographic imaging			
Positron emission tomography (PET)-CT	118 (96.7)	82 (95.3)	36 (100.0)
CT	4 (3.3)	4 (4.7)	0 (0.0)
Treatment			
Concurrent chemoradiation (CRT)	55 (45.1)	32 (37.2)	23 (63.9)
Induction chemotherapy + CRT	42 (34.4)	36 (41.9)	6 (16.7)
Sequential chemo and radiotherapy	15 (12.3)	11 (12.8)	4 (11.1)
Radiotherapy only	10 (8.2)	7 (8.1)	3 (8.3)
Total RT dose ≥ 60 Gy	113 (92.6)	77 (89.5)	36 (100.0)
Total RT dose > 54 Gy and <60 Gy	9 (7.4)	9 (10.5)	0 (0.0)

**Table 2:** PTV < 700 ccm vs. PTV ≥ 700 ccm patients in the T/N-exact matched cohort.

Parameter	PTV < 700 ccm	PTV ≥ 700 ccm
	N (%)	N (%)
Total	29	29
Age, Years		
≥65	21 (72.4)	24 (82.8)
<65	8 (27.6)	5 (17.2)
Gender		
Male	16 (55.2)	23 (79.3)
Female	13 (44.8)	6 (20.7)
T-stage		
1	5 (17.2)	5 (17.2)
2	6 (20.7)	6 (20.7)
3	9 (31.0)	9 (31.0)
4	9 (31.0)	9 (31.0)
N-stage		
0	5 (17.2)	5 (17.2)
1	3 (10.3)	3 (10.3)
2	11 (37.3)	11 (37.3)
3	10 (34.5)	10 (34.5)
Histology		
Squamous cell carcinoma (SCC)	14 (48.3)	12 (41.4)
Adenocarcinoma (AC)	13 (44.8)	13 (44.8)
Not otherwise specified (NOS)	2 (6.9)	4 (13.8)
Treatment		
Concurrent chemoradiation (CRT)	13 (44.8)	13 (44.8)
Induction chemotherapy + CRT	10 (34.5)	9 (31.0)
Sequential chemo and radiotherapy	3 (10.3)	4 (13.8)
Radiotherapy only	3 (10.3)	3 (10.3)
Total RT dose ≥ 60 Gy	28 (96.6)	28 (96.6)
Total RT dose > 54 Gy and <60 Gy	1 (3.4)	1 (3.4)
Patient Cohort		
Retrospective evaluation	21 (72.4)	21 (72.4)
Prospective evaluation	8 (27.6)	8 (27.6)

**Table 3:** Short review of literature.

Authors	Paper Name	Year	Results
Wiersma, T.G., et al.	Concurrent chemoradiotherapy for large-volume locally advanced non-small cell lung cancer	2013	The single-center, retrospective study included 121 NSCLC stage III patients treated with CRT between 2004 and 2011. Median follow-up for all patients was 37.6 months. Median OS and PFS were 15.7 and 11.6 months, respectively, OS for patients with PTV > 700 ccm was 14.5 vs. 26.5 months for PTV ≤ 700 ccm (*p* = 0.009).
Bradley, J.D., et al.	Standard-dose versus high-dose conformal radiotherapy with concurrent and consolidation carboplatin plus paclitaxel with or without cetuximab for patients with stage IIIA or IIIB non-small-cell lung cancer (RTOG 0617)	2015	The open-label randomized, two-by-two factorial phase 3 study included 166 patients with unresectable NSCLC stage III treated with CRT between 2007 and 2011. On univariate analysis, increasing values of GTV and PTV were associated with an increased risk of death. On multivariate analysis, PTV was among the factors predicting OS.
Bradley, J.D., et al.	Long-term results of RTOG 0617 trial: standard- versus high-dose chemoradiotherapy with or without Cetuximab for unresectable stage III non-small-cell lung cancer	2020	Long-term results of the RTOG 0617 trial have confirmed a small PTV as a prognostic factor for better OS in inoperable stage III NSCLC treated with concurrent CRT.
Present study	Association between planning target volume and patient outcome in inoperable stage III NSCLC treated with chemoradiotherapy	2020	The single-center, retrospective and prospective study included 122 NSCLC stage III patients treated with CRT between 2011 and 2018. Median follow-up for all patients was 41.2 months. Median OS and PFS were 20.9 and 7.1 months, respectively, median OS for patients with PTV > 700 ccm was 14.1 vs. 33.4 months for PTV ≤ 700 ccm (*p* = 0.025).

NSABP: National Surgical Adjuvant Breast and Bowel Projec, RTOG: the Radiation Therapy Oncology Group, GOG: the Gynecologic Oncology Group.

## Supplementary Materials

The following are available online at https://www.mdpi.com/2072-6694/12/10/3035/s1, Figure S1: (A). Overall survival (OS) in the PSM cohort by PTV < 700 ccm vs. ≥700 ccm. (B). Progression-free survival (PFS) in the PSM cohort by PTV < 700 ccm vs. ≥700 ccm. (C). Regional recurrence-free survival (RRFS) in the PSM cohort by PTV < 700 ccm vs. ≥700 ccm.
