# Supplementary material for: Association of Planning Target Volume with Patient Outcome in Inoperable Stage III NSCLC Treated with Chemoradiotherapy: A Comprehensive Single-Center Analysis
Source: Cancers (Basel). 2020 Oct 19;12(10):3035. doi: 10.3390/cancers12103035 (PMC7603086; doi:10.3390/cancers12103035)
Supplement: Supplementary file 1 [file cancers-12-03035-s001.pdf]

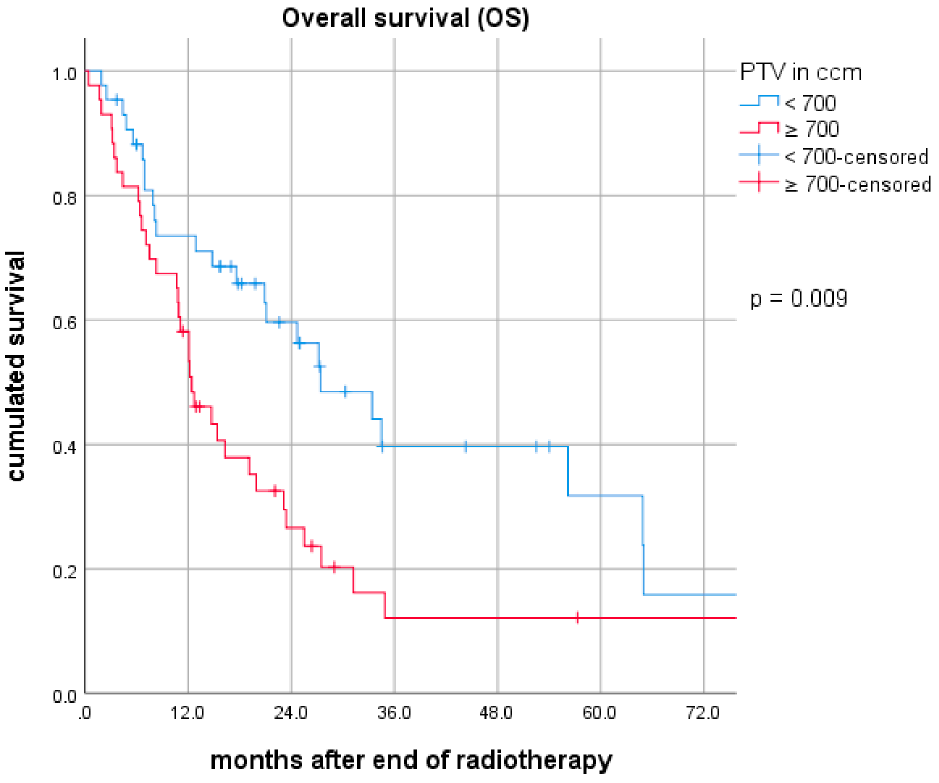
Overall survival (OS)
cumulated survival
months after end of radiotherapy
PTV in ccm
< 700
≥ 700
< 700-censored
≥ 700-censored
p = 0.009
1.0
0.8
0.6
0.4
0.2
0.0
.0
12.0
24.0
36.0
48.0
60.0
72.0

(**A**)

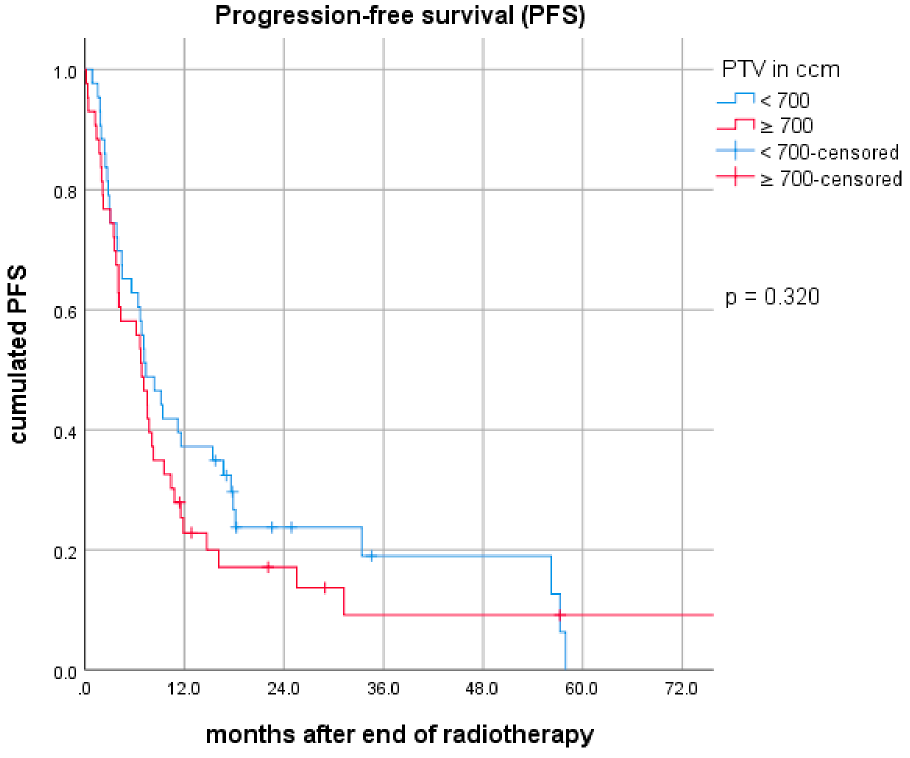
Progression-free survival (PFS)
cumulated PFS
months after end of radiotherapy
PTV in ccm
< 700
≥ 700
< 700-censored
≥ 700-censored
p = 0.320
1.0
0.8
0.6
0.4
0.2
0.0
.0
12.0
24.0
36.0
48.0
60.0
72.0

(**B**)

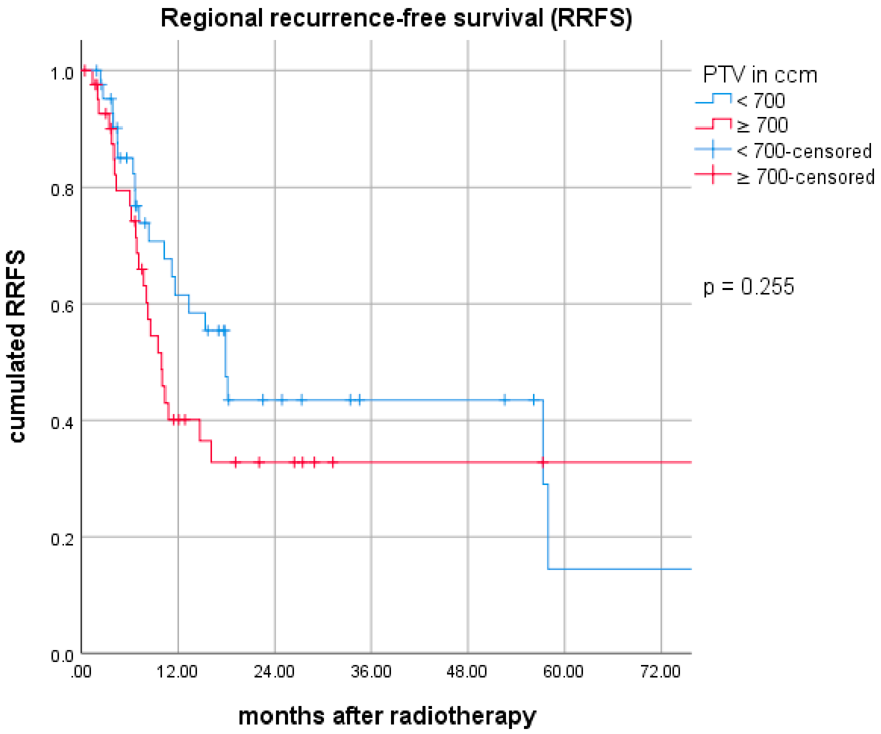

(C)

Figure S1. (**A**). Overall survival (OS) in the PSM cohort by PTV < 700 ccm vs. ≥700 ccm. (**B**). Progression-free survival (PFS) in the PSM cohort by PTV < 700 ccm vs. ≥700 ccm. (**C**). Regional recurrence-free survival (RRFS) in the PSM cohort by PTV < 700 ccm vs. ≥700 ccm.
